# Lattice Resonances and Local Field Enhancement in Array of Dielectric Dimers for Surface Enhanced Raman Spectroscopy

**DOI:** 10.1038/s41598-018-33941-7

**Published:** 2018-10-24

**Authors:** Jernej Černigoj, Fabrizio Silvestri, L. Pjotr Stoevelaar, Jonas Berzinš, Giampiero Gerini

**Affiliations:** 1 The Netherlands Organization for Applied Scientific Research, TNO, Optics Department, 2628CK Delft, The Netherlands; 2 Eindhoven University of Technology, TU/e, Electromagnetics Group, 5600MB Eindhoven, The Netherlands; 3 Friedrich Schiller University Jena, Institute of Applied Physics, Abbe Center of Photonics, 07745 Jena, Germany

## Abstract

In this paper, we propose the use of high refractive index dimers for the realization of a surface enhanced Raman spectroscopy substrate, with an average enhancement factor comparable to plasmonic structures. The use of low loss dielectric materials is favorable to metallic ones, because of their lower light absorption and consequently a much lower heating effect of the substrate. We combined two different mechanisms of field enhancement to overcome the main weakness of dielectric dimers: a low enhancement factor compared to the plasmonic ones. A first mechanisms is associated to surface lattice resonances. This generates a narrow-band high enhancement, which is exploited to enhance the excitation light. A second mechanism exploits the local field enhancement between the dimers’ resonators, for the band where the molecule Raman emission spectrum is located. The fact that both field enhancements can be tuned by acting on separate geometric parameters, makes possible to optimize the design for many different molecules. The optimized structure and its performance is presented together with a discussion of the different enhancement mechanisms.

## Introduction

In recent years, a lot of attention has been devoted to the development of reliable label-free molecule identification techniques^1^. These mechanisms can provide fast detection of a variety of molecules without prior need for labeling with fluorescent dyes and without bleaching problems. Thanks to this, cheap and fast sample analyses in medicine, personal health care, home-land security and environmental studies, could be achieved^1^. A promising candidate to achieve reliable label free molecular detection is Raman scattering. In this physical phenomenon, a photon interacts with the sample molecule and, at a particular wavelength, it excites vibrational modes of the molecule and loses part of its energy into this process. Spectral analysis of the scattered light provides the vibrational spectrum of the molecules under analysis. However, since the Raman cross section per molecule is typically low (10^−30^ cm^2^/molecule), the outcome of a Raman measurement is limited to a reduced number of in-elastically scattered photons^1^. To overcome this problem, Surface Enhanced Raman Spectroscopy (SERS), based on plasmonic nano structures, is usually employed. This can provide a significant signal enhancement, even to the point of single molecule detection^2–4^. Typically the total enhancement is the product of electromagnetic and chemical SERS enhancements. The first one occurs when the incident light interacts with surface plasmons in the metal. The second one is the result of a larger molecular cross section, due to a modification of its electronic structure near the metal^5^.

In case of Raman scattering, there is an enhancement for the excitation and for the emission light, in general, resulting in a total enhancement proportional to the fourth power of the field amplitude^5,6^. When the SERS substrate is not characterized by a uniform field enhancement over the wavelength band of interest, comprising excitation and emission spectra, the Enhancement Factor (EF) should be computed considering the field amplitudes at separate bands^5–8^.

SERS substrates are usually characterized by their EF, but there are many possible definitions of EF and this is also one of the reasons why they can vary over many orders of magnitude. Average EFs for plasmonic substrates are reported to be in the order of 10^4^–10^8^ ^2,7^.

Due to their high EF, plasmonic nanostructures are still mostly used for SERS substrates, but their large absorption coefficients encouraged researchers to look for alternative solutions^9^. This absorption poses many questions from the thermal point of view, especially due to the limited volume where such an absorption takes place. For this reason, a reduction of the absorption levels, by using not plasmonic material is of interest for the SERS community. Low loss and high refractive index dielectric materials have shown promising results and the potential to be used for SERS^10^. It has been shown through numerical simulations and also experimental validation that dimer structures made of high refractive index materials produce both electric and magnetic hotspots in infrared^11^ and in the visible spectral range^8,12^. The enhancement is produced by the displacement currents and the electric and magnetic dipole-dipole interactions in the dielectric dimers^11,13^. Dielectric dimers not only enhance the field, but they also provide control over the directivity of the far field radiation of the emitters, without quenching the nearby emitters and lowering their quantum efficiency^8^. However, the EF of a single dielectric dimer has been shown to reach levels consistently below the levels of plasmonic scatterers. Dimers placed in a periodic array were already studied and it has been shown that Surface Lattice Resonances (SLR) are present^14,15^. These resonances are narrow band and Fano line shaped. They provide higher field enhancement than a single dimer and also a beaming effect of the scattered light^14^. In this contribution, we will show that, by properly exploiting the SLR in a regular array of emitters, the dielectric dimers can be a valid replacement for plasmonic scatterers in field enhancement applications.

## Results

**Figure 1:**
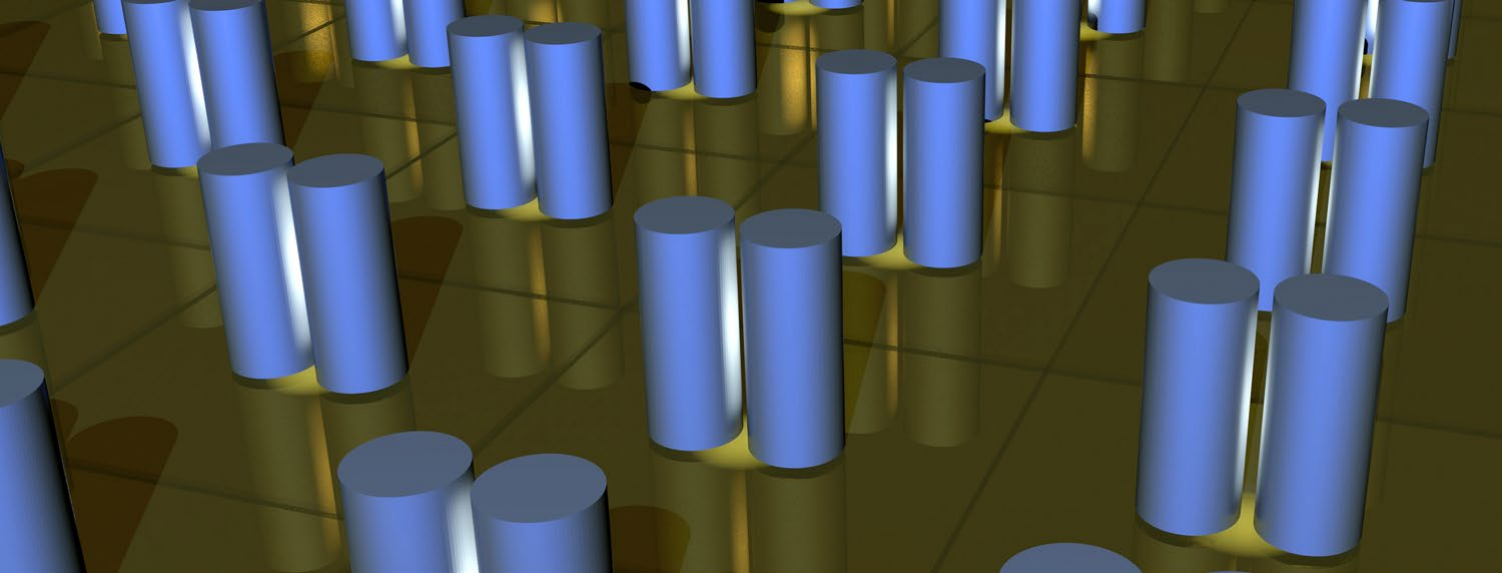
Artistic impression of a SERS substrate consisting of a rectangular periodic lattice of silicon dimers.

**Figure 2:**
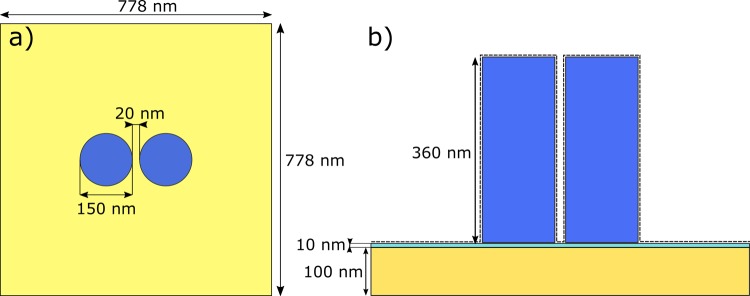
(**a**) Top view of a unit cell of the infinite array employed in numerical simulations. A dimer consists of two pillars separated by 20 nm gap. The dimer is illuminated with electric field polarized along the direction of separation of the two pillars. The two pillars are symmetrically displaced with respect to the center of the unit cell. (**b**) Side view of a unit cell. The substrate is made out of 100 nm thick layer of gold, on top of which there is a 10 nm thick SiO_2_ spacer. Also plotted in dashed line, the projection of the surface used for the integration of the average EF (see Methods).

In this section, we present the design and the performance of a SERS substrate, consisting of a periodic array of silicon dimers (Fig. 1). The layout with the dimensions of the array unit cell is reported in Fig. 2. The dimers are made of two pillars with a height of 360 nm and a diameter of 150 nm. The gap between the two pillars is 20 nm. The dimers are placed in an array with a square unit cell of 778 × 778 nm. The substrate is made of a 100 nm thick gold mirror on top of which there is 10 nm thick spacer of fused silica (SiO_2_). The above dimensions have been selected after a full investigation of the parametric landscape aimed at maximizing the field enhancement for an excitation wavelength of *λ* = 785 nm and emission wavelength in the neighborhood of *λ* = 850 nm. The design choices made during this optimization process were based on a-posteriori analysis of the simulation results. Their main implications are reported in the Supplementary Information. The low index SiO_2_ spacer is inserted to prevent the excitation of plasmonic waves at the interface between the dimers and the gold substrate, in a similar fashion to what was done for other reflective nanostructure-based substrates^16^. The refractive indices of the materials, used in the design, have been taken from literature^17–19^. When illuminated with a normally incident plane wave, with the electric field polarized along the axis of displacement of the dimers, a strong electric field enhancement between the two pillars is achieved. This field enhancement is a consequence of the interference between the incident light and the field scattered by the two pillars. The presence of a reflective substrate allows for a second interference for the forward scattered field, which otherwise would have been lost.

**Figure 3:**
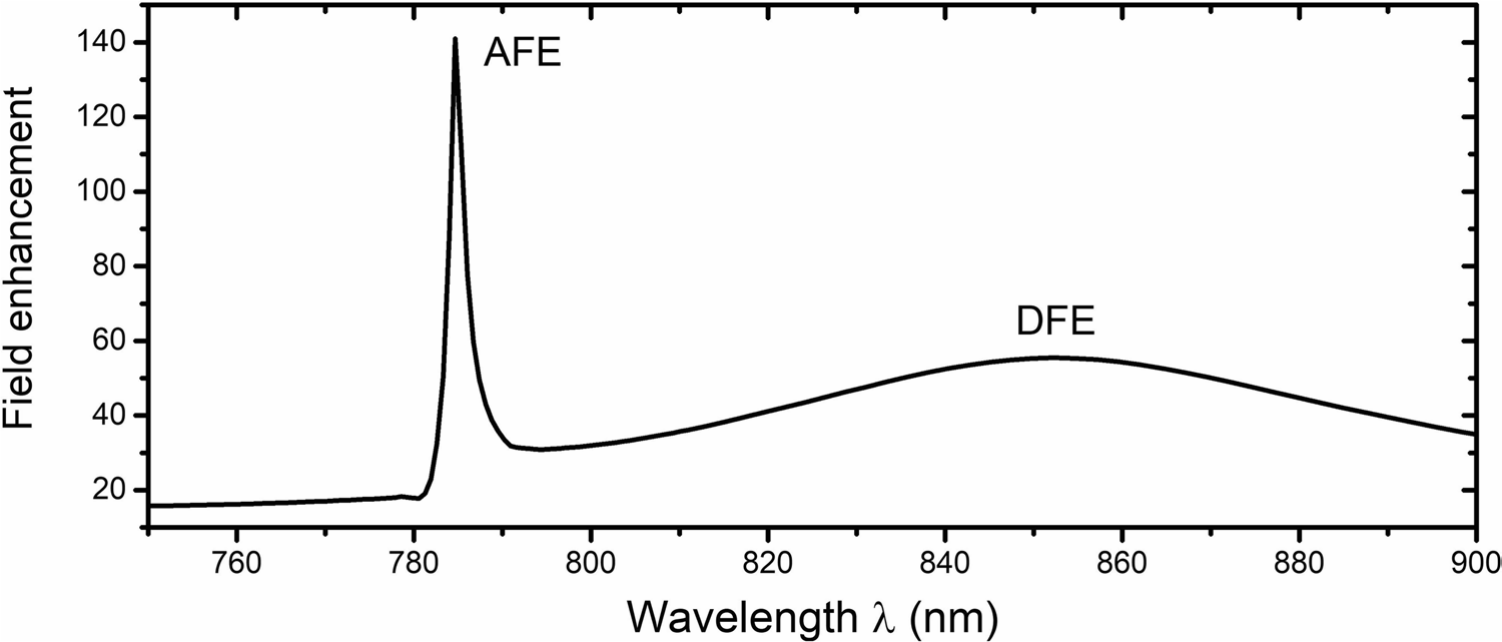
Maximum field enhancement along the longitudinal axis between the dimers with respect of wavelength. The AFE is located around *λ* = 785 nm with a FWHM = 3 nm while the DFE is located around *λ* = 850 nm and with FWHM = 90 nm.

The results of the numerical simulations of the array of dimers are reported in Fig. 3, where the maximum electric field enhancement within the gap is reported as function of the wavelength. From the plot, two spectral locations of enhancement can be identified. We call the first one the Array Field Enhancement (AFE). The AFE is positioned at around *λ* = 785 nm and it is characterized by a very high value of electric field enhancement (≈140) and a narrowband behavior (Full-Width Half Maximum, FWHM ≈ 3 nm). Several numerical simulations, whose results are not shown here for the sake of brevity, have confirmed that this peak originates from SLR. The spectral position of this peak, in fact, is mainly dictated by the value of the lattice period and marginally by the geometric dimensions of the dimers. Similar effects have been already noticed in periodic arrays of plasmonic antennas^15^ and in arrays of dielectric nanowires^14^. Nevertheless, according to our knowledge, this is the first attempt to combine the SLR with the local resonances of dielectric dimers to tailor an engineered substrate for SERS application. The proposed structure has been optimized to have the AFE located around 785 nm. This is an excitation wavelength often used in SERS experiments^20^.

The second peak, located around *λ* = 850 nm, has a spectral position mainly dependent on the geometric dimensions of the silicon pillars (diameter, height) and gap in-between^8,11,12^. For this reason, we label it Dimer Field Enhancement (DFE). The DFE is typically characterized by a smoother wavelength behavior (in this case FWHM = 90 nm) than the AFE. The fact that the spectral positions of AFE and DFE are almost geometrically uncoupled allows for the optimization of a substrate for SERS experiments. In fact, the two peaks can be separately tuned, one at the Raman excitation wavelength and the other one around the most populated range of Raman emission wavelengths (in this case: DNA molecules^21^, phosphate^22^). General guidelines for the spectral tuning of the AFE and DFE, by choosing the optimal geometric parameters, are reported in the Supplementary Information. The numerical simulations results (reported in Figs S4–S6) show that the AFE and DFE magnitudes depend on the distances of their spectral peaks. This effect brings us to the conclusion that there is an interference between these two resonances. It is therefore tempting to try to make the two peaks overlapping, to achieve the highest field enhancement. However, when the geometrical dimensions of the pillars have been modified to achieve this overlap, a sensible reduction of the AFE peak has been observed, suggesting a destructive interference, which is most likely due to the different spatial position of the AFE and DFE hotspots.

**Figure 4:**
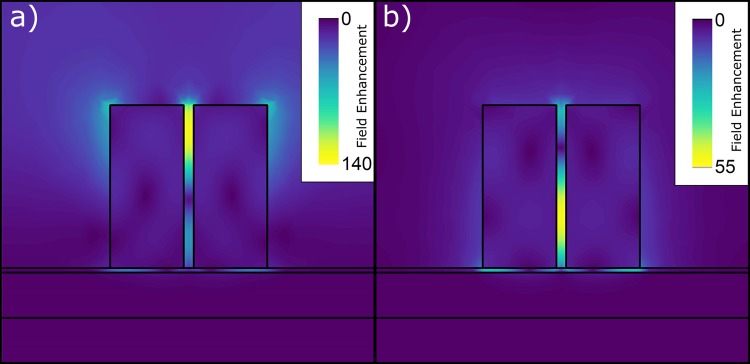
Field enhancement distribution at wavelengths of maximum AFE on the left (**a**) and maximum DFE on the right (**b**). The maximum enhancement in the case of AFE is at the top (314 nm from the dimer base) of dimer and for DFE maximum is more in the middle of the dimer (120 nm from the dimer bottom).

In Fig. 4, the electric field magnitude distribution is shown at the wavelengths of maximum AFE (a) and maximum DFE (b), respectively. From these graphs, it is evident that the zone with the highest electric field amplitude is located at the top of the dimers for the AFE, while at almost half height of the dimers for the DFE. This means that the molecules that will experience the highest excitation field will not have a comparable enhancement for emission wavelength. However, considering the average over a surface enclosing the dimers and the surrounding substrate, the field enhancement is still high. The spatial shift of the maximum field enhancement towards the top of the dimers is beneficial, because it prevents the formation of surface waves at the dimers base interface. This limits also the formation of plasmonic surface waves, with their related losses, which were already limited by the insertion of the silica spacer.

**Figure 5:**
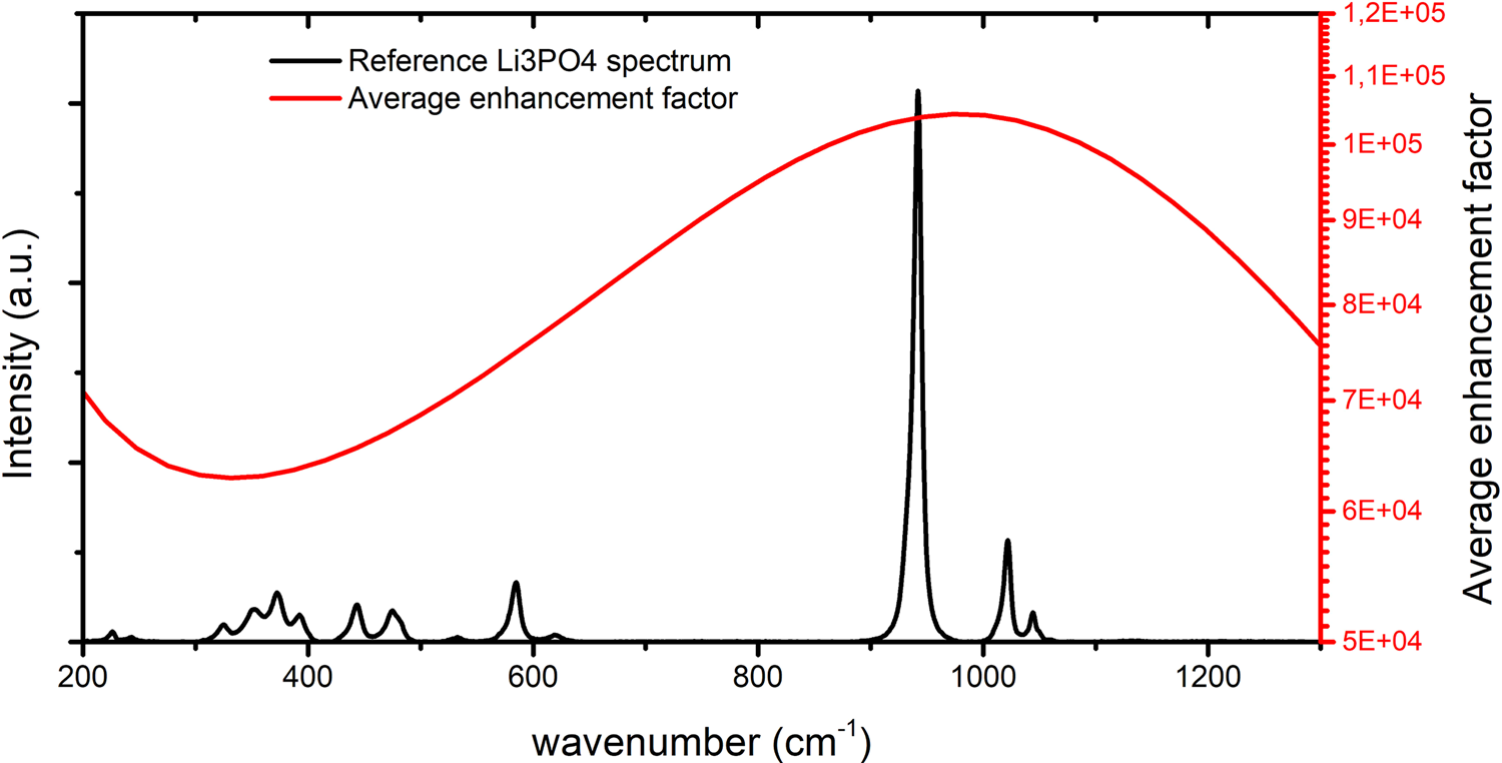
Average EF shown on top of lithium phosphate Raman spectrum. The spectrum was plotted from public spectral data available at the RRUFF Raman spectral database^24,25^. This spectrum has similar Raman emission lines to phosphate anion. Wavenumbers for EF were calculated with excitation wavelength of ≈785 nm. Enhancement changes from $6\cdot {10}^{4}$ to $1\cdot {10}^{5}$.

In the SERS community, the EF for Raman scattering due to electromagnetic enhancement mechanisms is typically computed as the fourth power of the maximum field amplitude^5^. This assumption comes from the usually employed approximation that the light intensity due to the structures surrounding the samples is almost equal at the excitation and emission wavelengths. While this works for most plasmonics based substrates^6^, which are characterized by broadband spectral response, this is not the case for the dielectric dimers, as it is shown in Fig. 3. Therefore, in order to get a better quantification of the EF, for the proposed structure, it is better to not use the previously mentioned approximation, but introduce a new definition (described in the Methods section). The EF, computed according to the new definition, is presented in Fig. 5. In this graph, the EF is superimposed to the Raman emission spectrum of lithium phosphate (${{\rm{Li}}}_{{\rm{3}}}{{\rm{PO}}}_{{\rm{4}}}^{{\rm{3}}}$), which is representative of other molecules like phosphate anion (${{\rm{PO}}}_{{\rm{4}}}^{{\rm{3}}-}$), DNA molecules. The plot shows that, for the emission peaks of the Li_3_PO_4_ molecule, an enhancement of at least ${\rm{EF}}\approx 6\cdot {10}^{4}$ is achieved, with a maximum of ${\rm{EF}}\approx 1\cdot {10}^{5}$ for the highest peak.

To estimate the EF, it is assumed that the sample molecules will be distributed randomly over the whole SERS substrate with silicon dimers. Only those molecules, which will end up in the zone with a high field enhancement, will contribute to the detected Raman signal. Performing an average of the field enhancement over a surface within the unit cell gives a better quantitative evaluation of the achievable Raman signal. This definition provides additional interesting insights in the Raman enhancement signal. In fact, for this specific design we have observed that by reducing the dimensions of the pillars it is possible to align the AFE and the DFE to have them overlapping. However, in that case the overall average EF is lower than what reported in this article. Therefore, the current definition of the EF is a useful tool for the design of Raman enhancement structures. The combination and the proper spectral tuning of the AFE and DFE provides an overall EF that is comparable to typical values of EF for plasmonics nanostructures^2,7,11^.

## Discussion

The use of two enhancement mechanisms, based on different geometric parameters, allows to control each enhancement separately and therefore to easily optimize a structure for different spectral ranges and different molecules. The AFE peak is typically narrowband, and consequently it is suitable to enhance the excitation light which is generated with mostly narrowband and stable lasers. Additionally, the spectral position of the AFE peak depends on the lattice dimensions, which can be controlled very precisely during manufacturing. On the other hand, the DFE peak spectral response is wider and therefore it can cover multiple emission lines. The control of the dimer dimensions is more challenging in the manufacturing process, however, the smoother spectral response of the DFE ensures that manufacturing tolerances will not have a significant effect on the total enhancement.

For this design, the structure has been optimized targeting an excitation wavelength of a standard Raman laser with *λ* = 785 nm and to the emission lines of the phosphate anion (${{\rm{PO}}}_{{\rm{4}}}^{{\rm{3}}-}$) molecules^20^. Nevertheless, a similar structure can be designed for a DNA molecule which has similar characteristics for the Raman spectrum^21^. In the case of phosphate molecules, an additional advantage of dielectric dimers has to be mentioned. Phosphate and other oxyanions have low affinity for gold and silver, which prevents them to stick to the surface. This makes plasmonics-based SERS substrates difficult to be used for their analysis^22^. On the contrary, owing to the completely dielectric nature of the surface interaction, this design perfectly suits this type of application.

**Figure 6:**
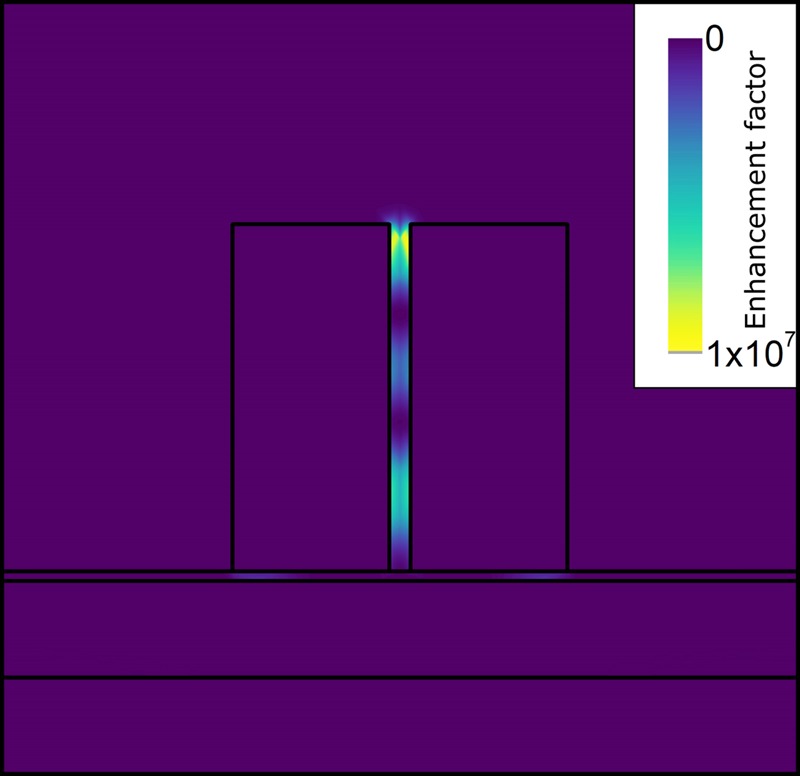
Spatial distribution of local EF, calculated for excitation wavelength of ≈785 nm and for emission wavelength of DFE maximum. The maximum EF is located at the top of dimer, with the maximum value ${{\rm{EF}}}_{{\max }}\approx 1\cdot {10}^{7}$. Beside hot spot at the top of dimer, there are two additional regions with high EF, located at the middle and close to the bottom of the dimer.

The EF computed in this paper is obtained through an averaging operation over the unit cell surface and it takes into account the different values of field enhancement at the excitation and emission wavelengths. As it can be seen in Fig. 4, the maximum AFE and DFE do not spatially overlap, and they are quite localized in narrow volumes within the gap of the pillars. Therefore, considering only the maximum ${{\rm{AFE}}}^{2}\approx {140}^{2}$ and ${{\rm{DFE}}}^{2}\approx {55}^{2}$ within the dimers will bring to an overestimated ${\rm{EF}}\approx 6\cdot {10}^{7}$. This level of EF is obviously not physical, since the molecule cannot be placed in a position where both maximums occur. A correct estimation of the EF requires to consider simultaneously the AFE and DFE at the each point in space. The product of the two spatially variable field enhancements, at the two different wavelengths, gives a maximum ${{\rm{EF}}}_{{\max }}\approx 1\cdot {10}^{7}$ (Fig. 6). This is localized in a small volume around the top of the dimers. With a control mechanism, which allows to place the molecule at exactly this position, this EF_*max*_ can be accessed with high repeatability. In a more general case, when this control mechanism is absent, we need to invoke an averaging operation.

A definition based on a spatial average over a surface enclosing the pillars and the neighboring substrate, Fig. 2, will lead to ${\rm{EF}}\approx 1\cdot {10}^{5}$. The mathematical formulation of this averaging process is reported in the Methods section. This value of EF is comparable to the one obtained with the plasmonic scatterers^2^ ${\rm{EF}}\approx 1\cdot {10}^{4}-{10}^{6}$. Higher values of average ${\rm{EF}}\approx 1\cdot {10}^{8}$ have been obtained with pairs of plasmonic inclusions separated by tiny gaps^7^. For the case of dielectric dimers, reduction of the gap provides also a boost of field enhancement. This is reported in the Supplementary Information in Fig. S6. However for this work we fix the gap to 20 nm, since this is a good compromise between high field enhancement and difficulty of fabrication. To get these gaps dedicated nanofabrication techniques must be employed^8,10^. In our computation of the EF, any effect on molecule orientation is neglected. In this sense, the EF here computed must be interpreted as the highest accessible EF.

## Conclusion

In this contribution, we have presented the design of an array of silicon dimers to be used as a SERS substrate. By a properly exploiting the AFE and DFE spectral peaks, the EF can be maximized up to levels of around 10^5^, which are close to the values of the typical metallic nanostructures, commonly employed in SERS experiments. The exploitation of the lattice resonance provides an additional strong peak, the AFE, which allows to obtain an EF larger than for typical structures employing dielectric dimers^8,11,12^. Moreover, field enhancement hotspots are located far from the gold substrate, and so issues related to the heating typical of plasmonic nanostructures are avoided^21^. This allows the use of higher intensity laser. The presence of the gold reflector provides an additional path for the interference between the incident and the scattered field, which brings to a higher field enhancement. These results pave the way for a more extended use of dielectric dimers array for SERS experiments.

## Methods

### Simulations of the periodic array

The Finite Element Method electromagnetic commercial software Ansys HFSS has been used to perform the numerical simulations^23^. The simulation model consists of one unit cell with one dimer (two pillars). Top and bottom surfaces of the simulation volume are defined as Floquet ports, invoking periodic boundary conditions. All propagating modes within the unit cell have been used. The electric field maps, computed within the unit cell at different wavelengths, have been used to compute the enhancement parameters.

### Computation of the SERS enhancement factor

The method proposed for the calculation of the EF accurately represents the situation of experimental measurements. It is assumed that the sample molecules are deposited on the substrate surface. That can be done by drying a solution of water and sample molecules. It can be expected that the molecules would be uniformly distributed over the whole surface. Parts of these molecules would end up far from the dimers, but some of them would be attached to the sides of the pillars and positioned inside the gap. The ones inside the gap would experience a strong field enhancement and would also contribute the most to the total signal. To represent this distribution, we have generated equally spaced (1 × 1 nm) points at 1 nm away from model surface (dashed line in Fig. 2b). Then we have calculated the EF, averaged over the whole surface, according to Equation (1):

1 $$EF({\lambda }_{emis})=\frac{1}{A}{\int }_{A}{(\frac{{E}_{i}({\lambda }_{excit},{\bf{r}})}{{E}_{0}})}^{2}\times {(\frac{{E}_{i}({\lambda }_{emis},{\bf{r}})}{{E}_{0}})}^{2}d{\bf{r}},$$

where *A* is the surface enclosing the dimers (Fig. 2) and *E*_0_ is the electric field magnitude of the incident light. In Equation (1), we have averaged the product of the electric field intensity enhancement at the excitation wavelength (*λ*_*excit*_ ≈ 785 nm) and the electric field intensity enhancement at the emission wavelength *λ*_*emis*_. The EF is shown in Fig. 5. The choice for a surface integral rather than a volume integral within the cell is dictated by the fact that sample molecules will stick to the surface during Raman measurements.

## Electronic supplementary material

Supplementary Information

## Data Availability

The datasets generated and analyzed during the current study are available from the corresponding author on reasonable request.
