# Supplementary material for: Lattice Resonances and Local Field Enhancement in Array of Dielectric Dimers for Surface Enhanced Raman Spectroscopy
Source: Sci Rep. 2018 Oct 24;8:15706. doi: 10.1038/s41598-018-33941-7 (PMC6200810; doi:10.1038/s41598-018-33941-7)
Supplement: Supplementary file 1 — Supplementary Information [file 41598_2018_33941_MOESM1_ESM.pdf]

Supplementary information for Lattice Resonances and Local Field Enhancement in Array of Dielectric Dimers for Surface Enhanced Raman Spectroscopy

Jernej Černigoj, Fabrizio Silvestri, L. Pjotr Stoevelaar, Jonas Berzinš, Giampiero Gerini

General design guidelines

As mentioned in the main text, the presented nano patterned substrate can be optimized for several molecules with different Raman spectra. This means that we will need to shift the DFE peak to the appropriate spectral position, so we will have the maximal Enhancement Factor(EF) at the spectral position of molecules Raman emission lines. We have already observed that the spectral position of the DFE is dependent only on the pillars dimensions (diameter, D, and height, h). For this reason we have simulated a single dimer not placed in an array. The results of these simulations for different values of diameter and height are shown in Fig. S1,S2. The DFE shifts to the longer wavelengths with increasing pillar dimensions. This gives us several combinations of pillars dimensions for the same spectral position of the DFE peak. To get the best combination, we checked which aspect ratio ($R = h/D$), between height and diameter, gives us the highest enhancement. From Fig. S3, we see that a higher ratio gives us a higher enhancement. However, there is an upper limit for the field enhancement, when the aspect ratio reaches the value $R = 2.4$. A further increase in the aspect ratio has no significant benefit for a higher enhancement. For a finer tuning of the spectral position, only one dimension of the pillars, either D or h, needs to be varied, since an optimal amplitude of the DFE can be found keeping a constant aspect ratio. The tunability of the DFE peak is shown on Fig. S4, where the spectral displacement of the DFE is approximately 25 nm for each step of $\Delta D = 5$ nm and $\Delta h = 12$ nm.

The position of the AFE in the spectrum is determined by the lattice period, a. However, there is a residual coupling of the AFE with the pillars dimensions. This means that, as the DFE moves, also the AFE shifts in the spectrum, as seen in Fig. S4. Nevertheless, this movement can be compensated by acting on the lattice period, which is the main driving geometric parameter for the spectral location of the AFE (Fig. S5). The AFE will spectrally shift to longer wavelengths with increasing lattice period a. By comparing the results in Fig. S3 and Fig. S5, it can be seen that the DFE is higher when the dimers are inserted in an array. This comparison demonstrates that single dimer simulations can be employed to optimize the dimensions of the pillars with respect to the DFE, also when the dimer is finally inserted in an array. It can also be noticed, that as the peaks move further apart from each other, their amplitude is reduced. This indicates that both peaks experience some constructive coupling with each other when they are spectrally close. It is tempting to try to exploit this constructive coupling by overlapping the AFE and DFE to get an overall improvement in the amplitude. However, as in the case of the design with $D = 145$ nm and $h = 350$ nm, the AFE disappears and the DFE is almost not modified. In this condition, it is observed that the Surface Lattice Resonances (SLR) are suppressed or, at least, they do not provide any enhancement with respect to the DFE.

In a similar way, by varying the diameter and the height of the pillars and the lattice constant, the spectral position of the field enhancement can be moved to other parts of the visible and IR spectra. In this case, it might be necessary to chose a different dielectric material to maintain a low extinction coefficient at the wavelengths of interest.

On Fig. S6, we report the dependence of the field enhancement, with respect to the size of the gap between the pillars. We observe that the field enhancement is higher for smaller gaps, for both AFE and DFE. This was expected, since it was already reported for the DFE[1]. Furthermore, we have observed also that by changing the gap, the DFE and AFE move spectrally. However, the tunability of the spectral position by means of gap variation is not desirable, because also field enhancement is significantly changed. For this reason, the gap dimensions are chosen only on the basis of maximization of the field enhancement level at the peaks. The natural choice would be the smallest gap, however a limit is posed by the manufacturability of the pillars. Previous works with pillars of similar dimensions[2,3] have shown the feasibility of 20 nm gap between pillars.

Through all the article, the exciting field is assumed as a plane wave normally incident on the substrate, with the electric field polarized along the axis of displacement of the pillars. However, it is interesting to analyze the behavior of the structure under different polarization states. In fact, this provides a first evaluation on how much the polarization state should be aligned to the pillars to achieve sufficient enhancement performance. In Fig. S7 the field enhancement vs the wavelength is reported for different angles between the electric field and the displacement axis. The results show that both DFE and AFE reduce for larger angles. This is expected because, for larger polarization rotation angles, the scattered field contributing to the field enhancement will be just the component of the incident light, with the polarization along the axis of displacement of the pillars.

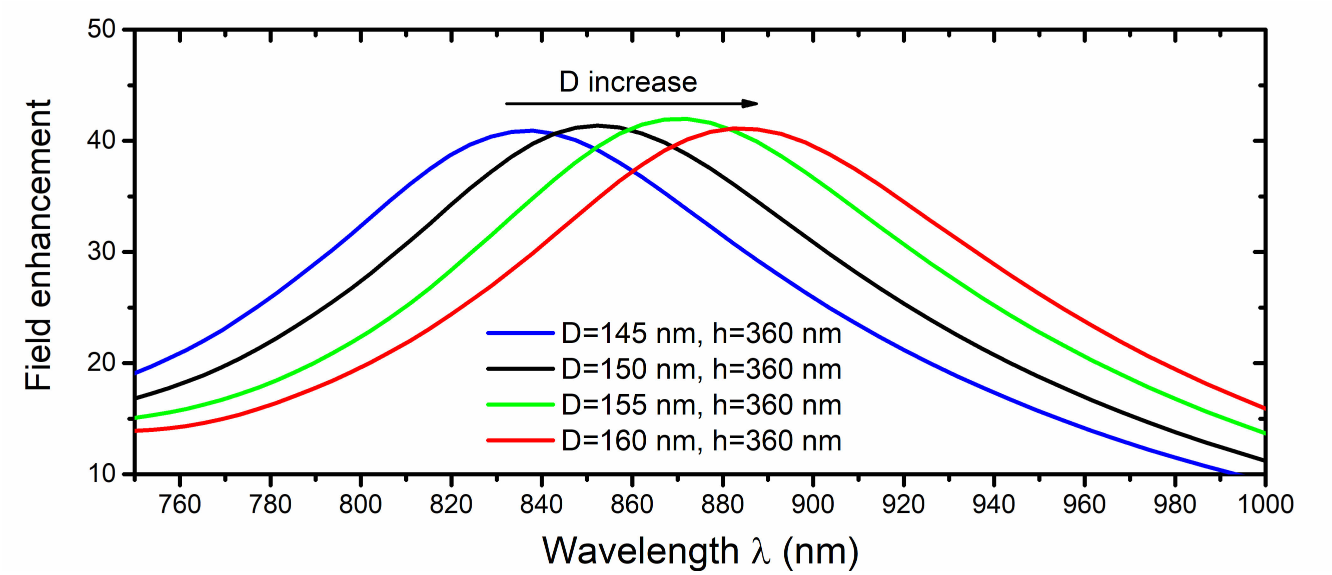

Figure S1. The maximum field enhancement along the longitudinal axis between the dimer with respect of the wavelength, for four different single dimers. Diameter, *D*, of the pillars is varied in steps of 5 nm. The maximum of the DFE peak moves towards longer wavelengths with respect to increasing diameters of the pillars. For each step of *D*, the spectral shift is approximately 20 nm.

References

1. Albella, P. *et al.* Low-loss electric and magnetic field-enhanced spectroscopy with subwavelength silicon dimers. *The J. Phys. Chem. C* **117**, 13573–13584 (2013).
2. Cambiasso, J., König, M., Cortés, E., Schlücker, S. & Maier, S. A. Surface-enhanced spectroscopies of a molecular monolayer in an all-dielectric nanoantenna. *ACS Photonics* **5**, 1546–1557 (2018).
3. Caldarola, M. *et al.* Non-plasmonic nanoantennas for surface enhanced spectroscopies with ultra-low heat conversion. *Nat. Commun.* **6**, 7915 (2015).

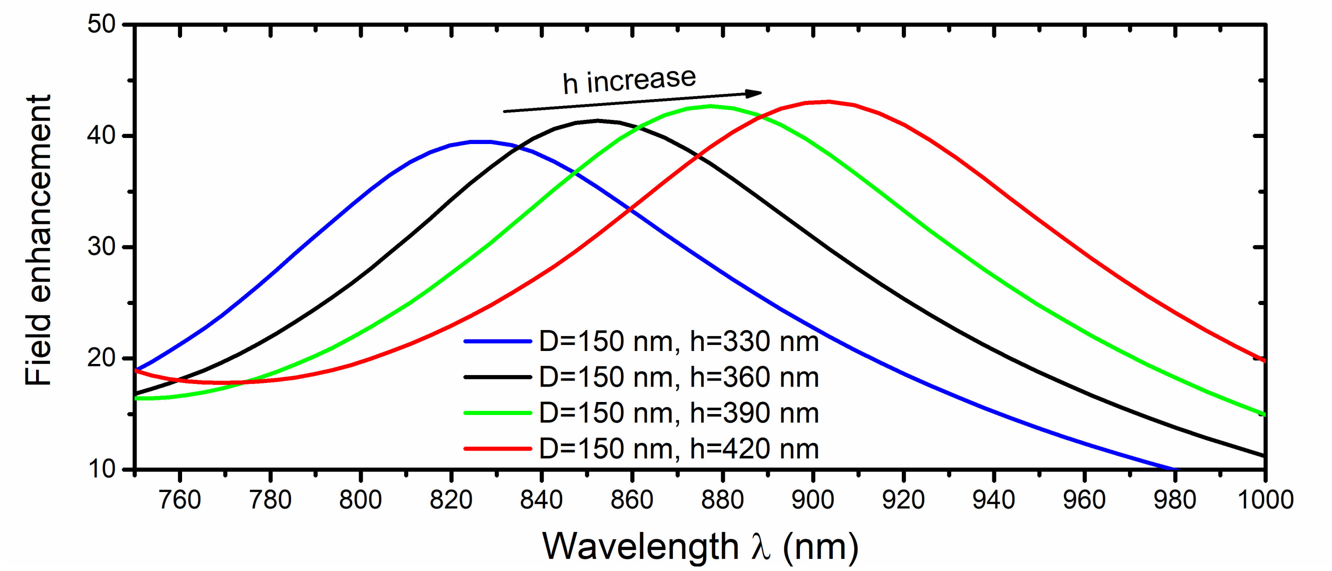

Figure S2. The maximum field enhancement for four different heights of single dimer. Height, h, of the pillars is varied in steps of 30 nm. The maximum of the DFE peak moves towards longer wavelengths with respect to increasing heights of the pillars. The maximum field enhancement increases with taller pillars.

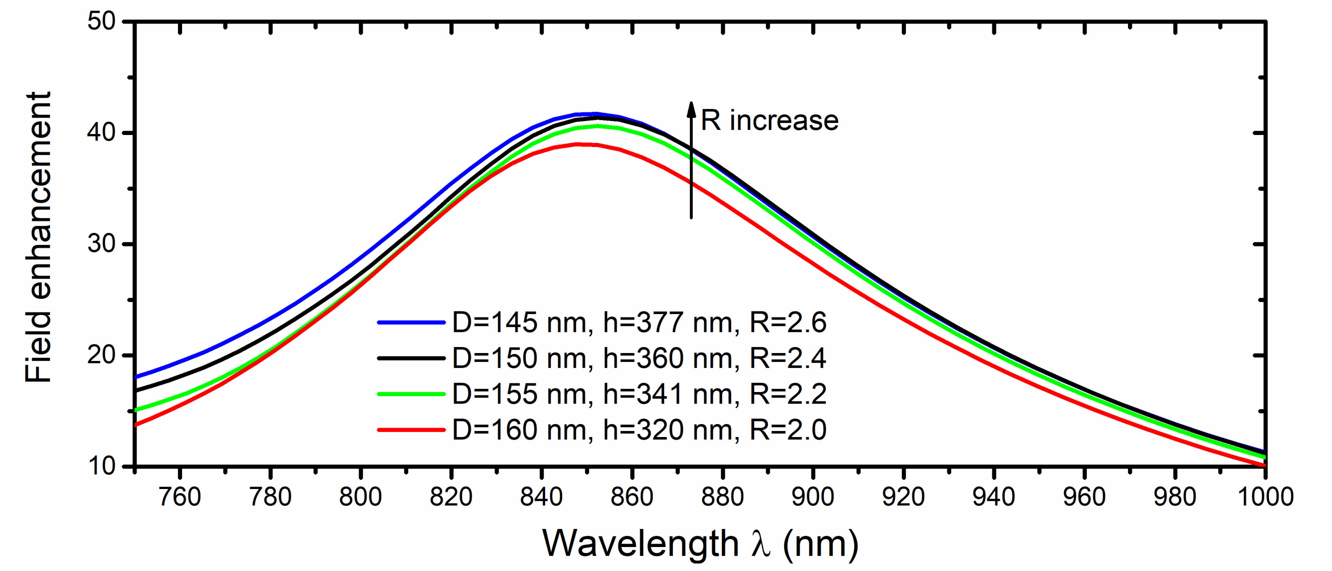

Figure S3. Optimization of the aspect ratio of the pillars. Here there are only shown those combinations of D and h which allows to have the DFE located at $\lambda \approx 850$ nm. The field enhancement increases with increasing ratio, up to an upper limit of $R = 2.4$.

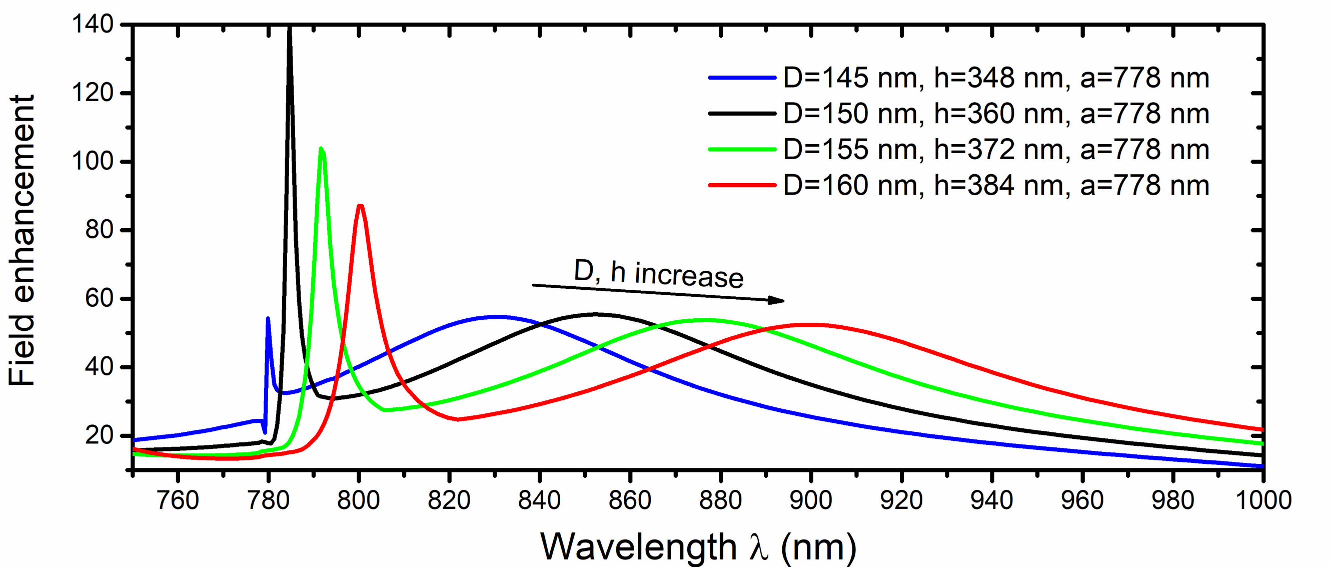

Figure S4. The maximum field enhancement along the longitudinal axis between the dimers with respect of the wavelength, for four different dimensions of substrate structures. Diameter, D, and height, h, of the pillars is varied in steps of 5 nm and 12 nm, respectively. The aspect ratio R is kept constant at value $R = 2.4$. With the change in pillars dimensions DFE and AFE peaks are spectrally shifted to longer wavelengths. This indicates that AFE is not completely uncoupled from pillars dimensions.

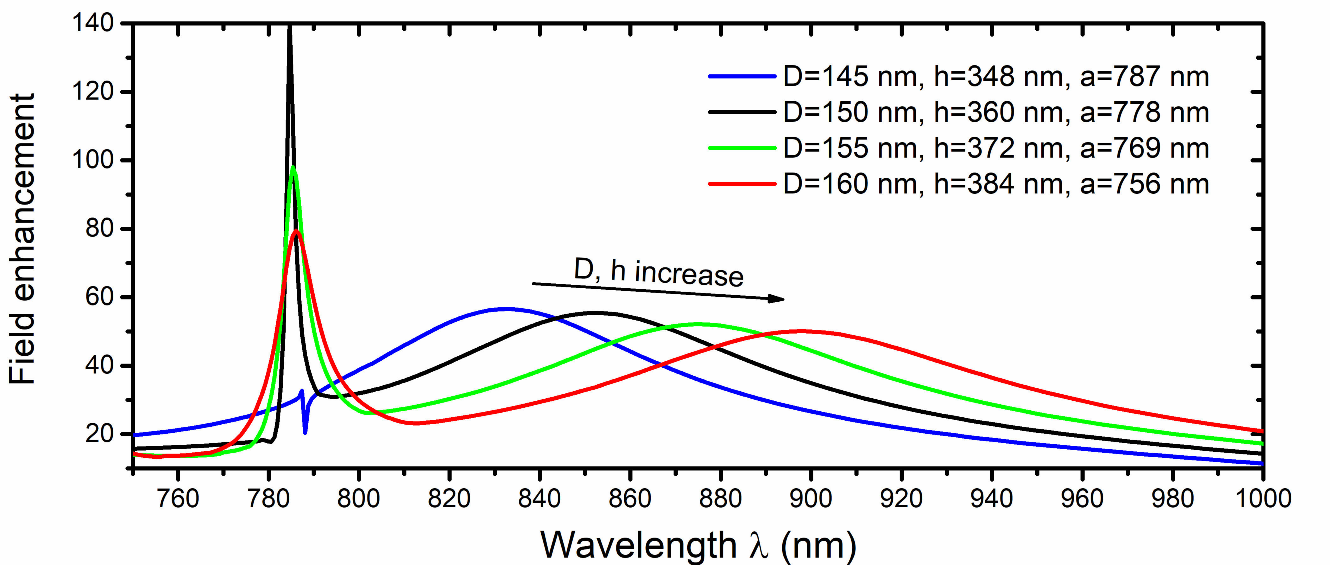

Figure S5. The maximum field enhancement spectra for four different dimensions of substrate structures. Diameter, D, and height, h, are varied in steps of 5 nm and 12 nm, respectively, as in Figure S4. The array lattice constant, a, is modified to compensate for the residual coupling of the AFE with the pillar dimensions, in order to maintain the AFE at almost the same spectral position.

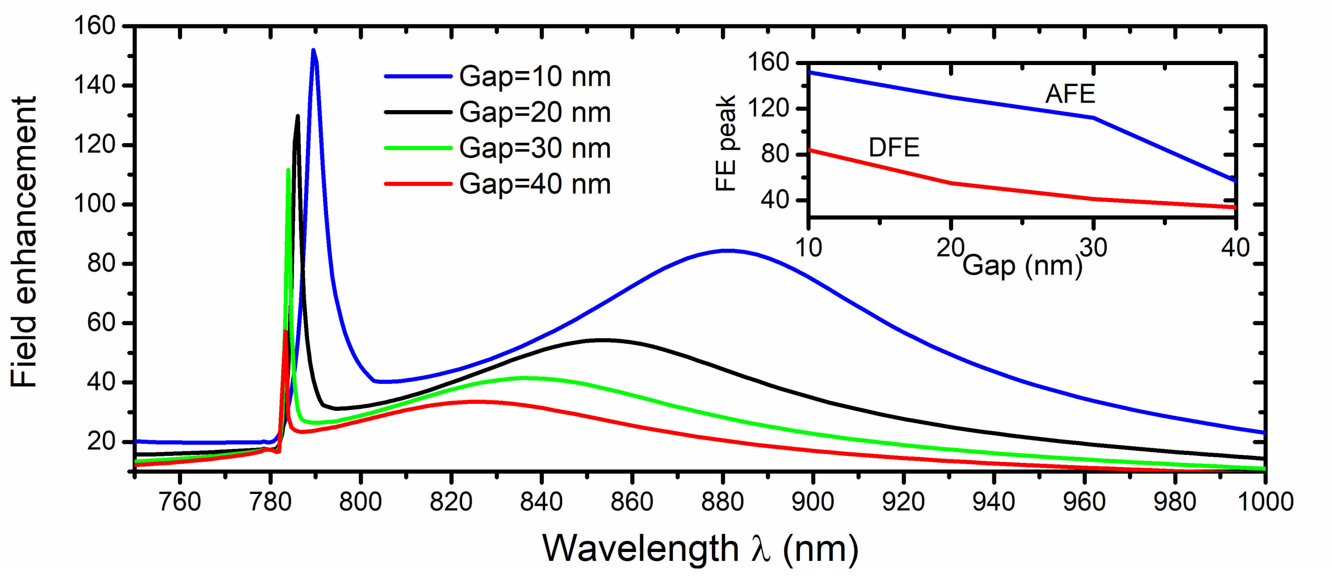

Figure S6. The maximum field enhancement spectra in cases of four different sizes of a gap between pillars. The field enhancement is higher for smaller gap. Both peaks move spectrally to longer wavelengths for smaller gaps. Insert: The amplitude of the DFE and AFE peaks with respect to the gap size.

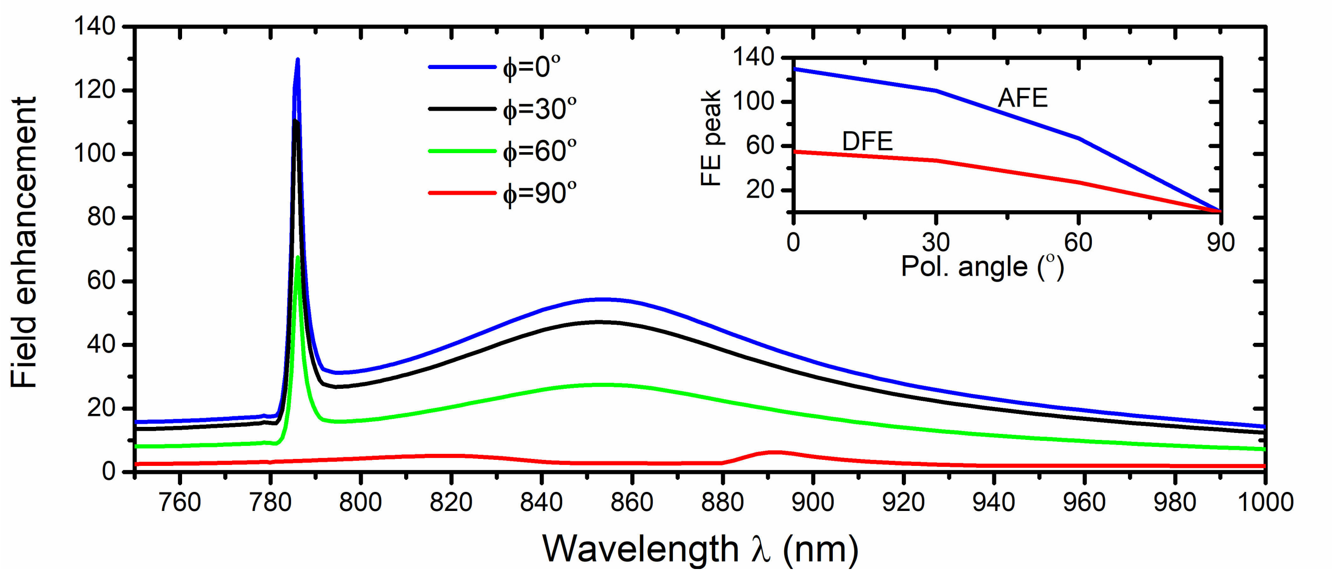

Figure S7. The maximum field enhancement spectra in cases of four different polarizations of incident light. $\phi = 0^\circ$ equals polarization in direction of displacement of the pillars. Field enhancement is highest when the light is polarized in direction of displacement and is failing when polarization angle is increased. For an angle $\phi = 90^\circ$ there is almost no enhancement. Insert: The amplitude of the DFE and AFE peaks with respect to the polarization angle.
